# Clinical Severity of SARS-CoV-2 Variants during COVID-19 Vaccination: A Systematic Review and Meta-Analysis

**DOI:** 10.3390/v15101994

**Published:** 2023-09-26

**Authors:** Zhilu Yuan, Zengyang Shao, Lijia Ma, Renzhong Guo

**Affiliations:** 1 School of Architecture and Urban Planning, Research Institute for Smart Cities, Shenzhen University, Shenzhen 518060, China; yuanzl13@szu.edu.cn (Z.Y.); guorz@szu.edu.cn (R.G.); 2 College of Computer Science and Software Engineering, Shenzhen University, Shenzhen 518060, China; zyshao0302@gmail.com

**Keywords:** COVID-19, SARS-CoV-2, variants, severity, systematic review, meta-analysis

## Abstract

Due to the variation in the SARS-CoV-2 virus, COVID-19 exhibits significant variability in severity. This presents challenges for governments in managing the allocation of healthcare resources and prioritizing health interventions. Clinical severity is also a critical statistical parameter for researchers to quantify the risks of infectious disease, model the transmission of COVID-19, and provide some targeted measures to control the pandemic. To obtain more accurate severity estimates, including confirmed case-hospitalization risk, confirmed case-fatality risk, hospitalization-fatality risk, and hospitalization-ICU risk, we conducted a systematic review and meta-analysis on the clinical severity (including hospitalization, ICU, and fatality risks) of different variants during the period of COVID-19 mass vaccination and provided pooled estimates for each clinical severity metric. All searches were carried out on 1 February 2022 in PubMed for articles published from 1 January 2020 to 1 February 2022. After identifying a total of 3536 studies and excluding 3523 irrelevant studies, 13 studies were included. The severity results show that the Delta and Omicron variants have the highest (6.56%, 0.46%, 19.63%, and 9.06%) and lowest severities (1.51%, 0.04%, 6.01%, and 3.18%), respectively, according to the four clinical severity metrics. Adults over 65 have higher severity levels for all four clinical severity metrics.

## 1. Introduction

Coronavirus disease 2019 (COVID-19) is an infectious disease caused by severe acute respiratory syndrome coronavirus 2 (SARS-CoV-2), which was first identified in Wuhan, China in late December 2019. As of 31 December 2022 [1], more than 660 million people have been confirmed to have COVID-19 and 6.69 million people have died from COVID-19 worldwide [2]. Unprecedentedly, more than 200 vaccines have been developed to end the epidemic, but the virus is also mutating [3]. Worldwide, five variants of concern (including Alpha, Beta, Delta, Gamma, and Omicron) have been identified by the WHO. However, the transmission rates and clinical severity of the variants have differences across different countries. For example, in France, there was a significant difference in the confirmed case-hospitalization risks between Delta (11.93%) and Omicron (1.34%) [4].

The overwhelming influx of COVID-19-infected patients to many hospitals has increased the burden on the healthcare system and even caused some medical systems to collapse in some areas [5,6]. An accurate estimation of clinical severity for COVID-19 is crucial for prioritizing interventions by governments (such as social distancing limitations, school closures, bans on large gatherings and nonessential activities, stay-at-home orders, travel restrictions, face masks, extensive mass testing, contact tracing, and isolation programs), the allocation of healthcare resources (including masks, ventilators, hospital beds, ICU support, and other medical equipment), and disease monitoring and surveillance [7,8,9,10]. For public health researchers and offices, severity is a critical parameter to model the transmission of disease and forecast the numbers of infections, severe illnesses, and deaths. Although several studies have evaluated the clinical severity of the COVID-19 disease, the reported clinical severity of the variants across studies has significant differences in different countries. For example, for the Delta variant, the confirmed case-hospitalization risk, cCHR, in Qatar [11] is 27.27%, while it is 1.34% in Norway [12].

Our objective was to summarize the clinical severity of the COVID-19 disease caused by different SARS-CoV-2 variants during mass vaccination, investigate severity differences in various countries and regions, and further estimate the impact of COVID-19 vaccination programs on the clinical severity of the COVID-19 disease. We conducted a systematic review and meta-analysis on the clinical severity of SARS-CoV-2 variants, and provided pooled estimates for each variant.

## 2. Methods

### 2.1. Measures of Clinical Severity of COVID-19 Disease

The clinical severity of an infectious disease is usually measured by indicators of confirmed case-hospitalization risk (cCHR), confirmed case-fatality risk (cCFR), hospitalization-fatality risk (HFR), and hospitalization-ICU risk (HIR) [7,9,13,14]. cCHR and cCFR denote the proportions of confirmed cases who were hospitalized and died, respectively. HFR and HIR denote the proportions of hospitalized cases who died and were in the intensive care unit (ICU), respectively.

### 2.2. Search Strategy and Selection Criteria

We conducted a systematic review to identify population-based studies reporting laboratory-confirmed COVID-19 cases during COVID-19 mass vaccination, following the Preferred Reporting Items for Systematic Review and Meta-Analyses guidelines. All searches were carried out on 1 February 2022 in PubMed for studies published from 1 January 2020 to 1 February 2022. We included all relevant studies published in peer-reviewed journals. Search terms for severity for COVID-19 variants included (#1) “severity” OR “fatality” OR “mortality” OR “death” OR “hospital admission” OR “ICU admission”; (#2) “COVID-19” OR “SARS-CoV-2”; and (#3) “variant” OR “mutation” OR “lineage” OR “amino acid substitution”; and the final search term was #1 AND #2 AND #3.

All studies were double-screened by two authors (Z.Y. and Z.S.) based on the titles and abstracts. Conflicts over inclusion of the studies were resolved by other coauthors (L.M. and R.G.). Studies were excluded if they were (1) not about COVID-19 variants; (2) the results from animal studies; (3) policies or modeling studies; (4) virology, genome, or protein studies; (5) infection and transmission studies; (6) detection and sequence analysis studies; (7) pathology or immunology studies; (8) treatment drug or program studies; (9) reviews, news, or case reports. All identified full-text articles were reviewed by Z.Y. and Z.S., and those with confirmed cases exceeding 100 and including multiple age groups were included.

### 2.3. Data Extraction

Data were extracted from each study by two authors (Z.Y. and Z.S.) independently. The data on clinical severity caused by SARS-CoV-2 variants during COVID-19 mass vaccination included the number of individuals diagnosed with COVID-19 and those who were hospitalized, were admitted to the ICU, or died. To better gauge the severity of various strains within a sizable populace while minimizing the impact of age and preexisting chronic illnesses, we excluded studies that did not pertain to the general population, i.e., those that included fewer than 100 confirmed cases across multiple age ranges. Other information, such as the study’s information (i.e., estimation period and location, vaccine information) was also extracted for each selected study (as shown in Table 1 and Table 2). For the reviewed articles with vaccine information, we collected the proportions of the general population and the elderly who received two doses of COVID-19 vaccine. For the reviewed articles without vaccine information, we collected vaccine information from the official website and “our world in data” according to the period and location, and then calculated the proportions of the population who received two doses of vaccine and the elderly who received two doses of vaccine.

### 2.4. Statistical Analysis

We calculated four clinical severities (cCHR, cCFR, HFR, and HIR) of COVID-19 coupled with the corresponding 95% CI based on the binomial distribution. For the overall effect of the different variants, we first used the I2 index to assess the heterogeneity of the included studies. The range of the heterogeneity statistic results was from 0 to 100%, in which I2 = 0–25% (no heterogeneity), I2 = 25–50% (moderate heterogeneity), and I2 > 75% (high heterogeneity). According to the I2 value calculated in the results and the significance of the Cochran Q test, a random-effects model or a fixed-effects model was further used to perform a meta-analysis in this study. In addition, meta-regression analysis using a mixed-effects model was conducted to quantify the association between the clinical severity and the population and elderly proportion with two doses of vaccines if the number of studies was near or above 10. All analyses were conducted in R version 4.1.2.

## 3. Results

**Figure 1:**
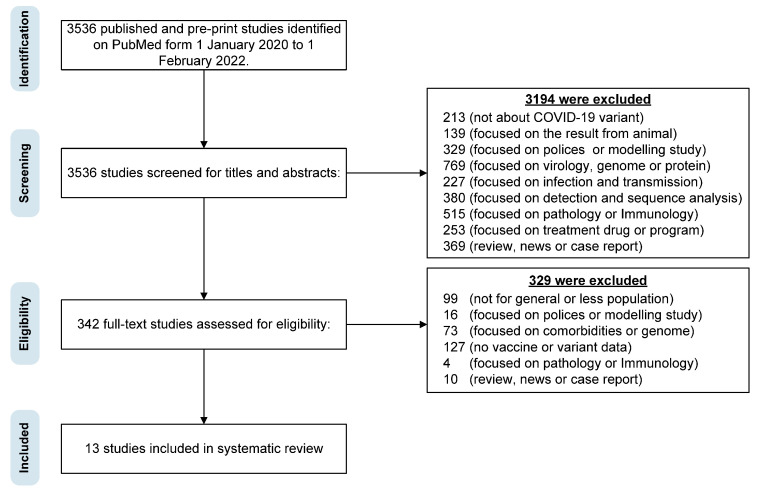
Preferred Reporting Items for Systematic Reviews and Meta-Analyses (PRISMA) flow diagram showing the screening process used to obtain included studies in the meta-analysis.

We identified a total of 3536 studies based on our search criteria. After excluding 3194 irrelevant studies based on their titles and abstracts and 342 studies based on their full text, 13 studies were finally included for meta-analysis. All our reviewed studies were from after patients received vaccination. The detailed selection process is illustrated in Figure 1, and the detailed characteristics of the included studies are summarized in Table 1. These studies were conducted in nine countries, namely the United States (the number of studies, *n*, is 2), the United Kingdom (*n* = 2), France (*n* = 2), Norway (*n* = 2), Israel (*n* = 1), Qatar (*n* = 1), Canada (*n* = 1), India (*n* = 1), and Denmark (*n* = 1). There are 21 sets of clinical results in our study, 5 for Alpha, 1 for Beta, 13 for Delta, and 2 for Omicron. However, two studies on Gamma in Brazil [29,30] were excluded because they only focused on the elderly, which means that no study on Gamma is included in Table 1.

**Figure 2:**
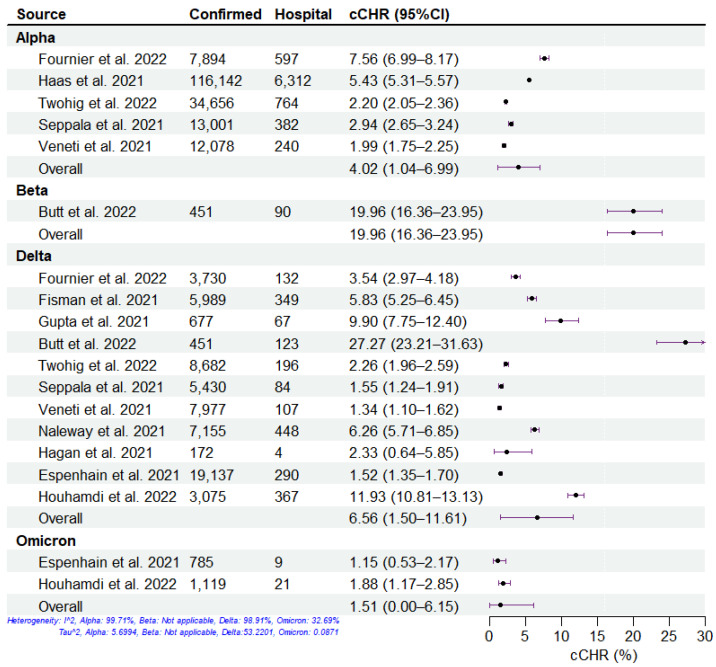
Estimation of confirmed case-hospitalization risk (cCHR) of SARS-CoV-2 variants. The cCHR of SARS-CoV-2 variants, including Alpha, Beta, Delta, and Omicron, from 12 studies. The dots and error bars demonstrate the estimated mean and 95% CI, respectively.

**Figure 3:**
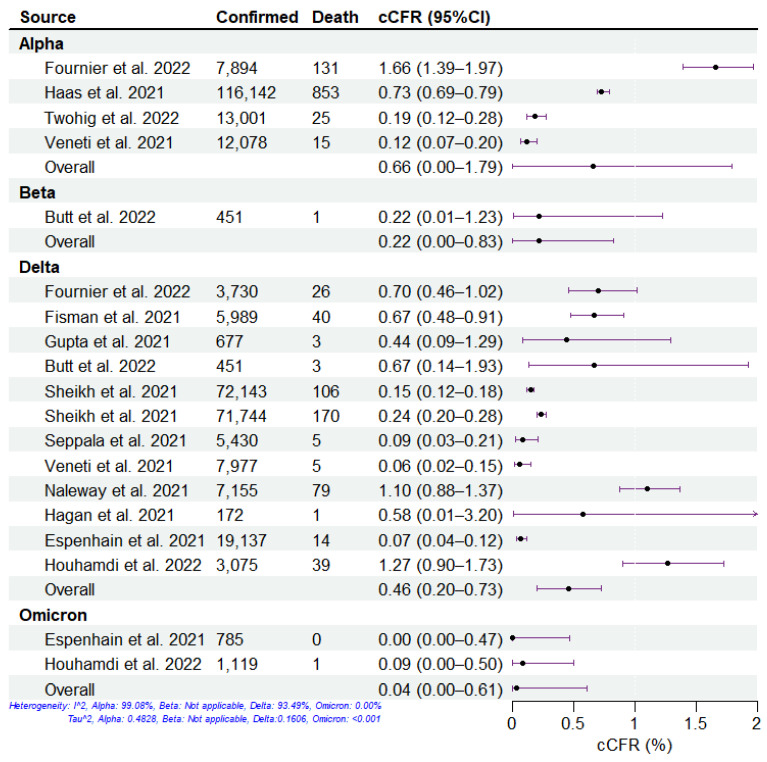
Estimation of confirmed case-fatality risk (cCFR) of SARS-CoV-2 variants. The cCFR of SARS-CoV-2 variants, including Alpha, Beta, Delta, and Omicron, from 12 studies. The dots and error bars demonstrate the estimated mean and 95% CI, respectively.

**Figure 4:**
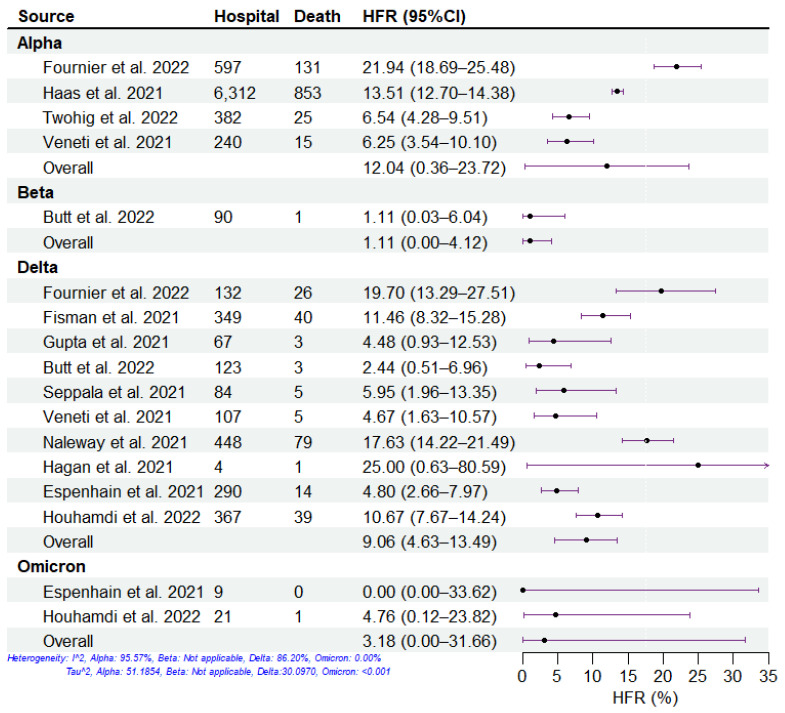
Estimation of hospitalization-fatality risk (HFR) of SARS-CoV-2 variants. The HFR of SARS-CoV-2 variants, including Alpha, Beta, Delta, and Omicron, from 11 studies. The dots and error bars demonstrate the estimated mean and 95% CI, respectively.

**Figure 5:**
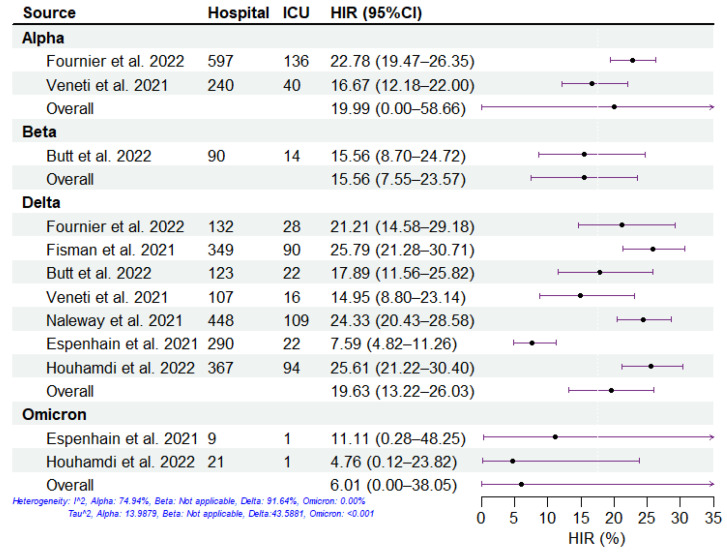
Estimation of hospitalization-ICU risk (HIR) of SARS-CoV-2 variants. The HIR of SARS-CoV-2 variants, including Alpha, Beta, Delta, and Omicron, from 7 studies. The dots and error bars demonstrate the estimated mean and 95% CI, respectively.

We summarized the clinical severity of different SARS-CoV-2 variants during the period of COVID-19 mass vaccination using the included studies (Figure 2, Figure 3, Figure 4 and Figure 5). Overall, across all four severities in the variants of concern, the HIR is the highest, followed by HFR, cCHR, and cCFR, which is consistent with reports about the principle of hospitalization (i.e., infected individuals with moderate or severe symptoms who are assessed to require hospitalization for treatment by a doctor are admitted to the hospital) [31,32]. For all SARS-CoV-2 variants, the Delta and Omicron variants have the highest and lowest severity, respectively, among the four clinical severity metrics. For the Beta variant, there was only one study included, which denoted high cCHR but low HIR and HFR, which may be attributed to sufficient healthcare conditions, successful public health policy, and government management measures in Qatar [33,34]. Specifically, the pathogen-specific cCHR ranged between 1.51% (95% CI: 0.00–6.15%; 2 studies) for Omicron, 4.02% (95% CI: 1.04–6.99%; 5 studies) for Alpha, 6.56% (95% CI: 1.50–11.61%; 11 studies) for Delta, and 19.96% (95% CI: 16.16–23.75%; 1 study) for Beta. The pathogen-specific cCFR ranged between 0.04% (95% CI: 0.00–0.61%; 2 studies) for Omicron, 0.22% (95% CI: 0.00–0.83%; 1 study) for Beta, 0.46% (95% CI: 0.20–0.73%; 11 studies) for Delta, and 0.66% (95% CI: 0.00–1.79%; 4 studies) for Alpha. The pathogen-specific HFR ranged between 1.11% (95% CI: 0.00–4.12%; 1 study) for Beta, 3.18% (95% CI: 0.00–31.66%; 2 studies) for Omicron, 9.06% (95% CI: 4.63–13.49%; 10 studies) for Delta, and 12.04% (95% CI: 0.36–23.72%; 4 studies) for Alpha. The pathogen-specific HIR ranged between 6.01% (95% CI: 0.00–38.05%; 2 studies) for Omicron, 15.56% (95% CI: 7.55–23.57%; 1 study) for Beta, 19.63% (95% CI: 13.22–26.03%; 7 studies) for Delta, and 19.99% (95% CI: 0.00–58.66%; 2 studies) for Alpha.

Besides the results for different variants, we further summarized the clinical severity by age group (Table 2). For studies with different age groups, we merged confirmed cases, hospitalized cases, and deaths into four age groups (0–24, 25–44, 45–64, and over 65). Similar to the calculation method in Table 1, the four types of severity were recalculated based on the number of cases coupled with the corresponding 95% CI based on binomial distribution. The results show that adults over 65 have higher severity levels in all four clinical severity metrics, perhaps due to their underlying medical conditions and some chronic diseases [35,36]. And younger people in the age group of 0–24 rank significantly lower than other groups in terms of the four clinical severities. For the Alpha variant pandemic in Norway, the confirmed case-hospitalization risk, cCHR, is 0.17% (95% CI: 0.09–0.31%) in the 0–24 age group, while it is 15.38% (95% CI: 11.22–20.35%) in adults over 65 [12].

Studies of clinical severity for the Delta variant were eligible for meta-regression analysis (Table 3). We used a mixed-effects model to study the relationship between population proportions with two doses of COVID-19 vaccine (Pf for all age groups; Po for the elderly over 65 years old) and clinical severity (cCHR, cCFR, HFR, and HIR), but did not find a significant relationship between them. This is perhaps due to the differences in healthcare conditions, vaccine strategies, and vaccine types among different countries and regions.

## 4. Discussion

The continuous emergence of new SARS-CoV-2 variants substantially increases uncertainty about the future. To investigate the existing evidence of clinical severity caused by different SARS-CoV-2 variants, we conducted a systematic review and meta-analysis of published studies that reported severity information for SARS-CoV-2 variants during the period of COVID-19 mass vaccination, including for Alpha, Beta, Delta, and Omicron, published between 1 January 2020 and 1 February 2022. The primary metrics of clinical severity were confirmed case-hospitalization risk (cCHR), confirmed case-fatality risk (cCFR), hospitalization-fatality risk (HFR), and hospitalization-ICU risk (HIR).

Among all SARS-CoV-2 variants, the highest clinical severity is observed in the HIR metric, followed by HFR, when compared with cCHR and cCFR. In terms of age groups, older adults over 65 have higher severity levels in all four clinical severity metrics. In addition, the Delta SARS-CoV-2 variant caused the highest severity for the four clinical severity metrics, while Omicron caused the lowest severity. However, only the first Omicron virus (BA.1) was considered in our study. The clinical severity of other Omicron viruses (such as BA.5, XBB, BQ.1, etc.) still needs further investigation. In terms of different countries, the cCHR in Qatar is 19.96% (16.36%, 23.95%) for Beta and 27.27% (23.21%, 31.63%) for Delta, which is the highest among the nine countries, but for the HFR and HIR, there is no significant difference from other countries, potentially due to the higher-quality healthcare conditions and hospital admission procedures in Qatar.

In early December 2020, the BNT162b2 mRNA vaccine first received a temporary emergency use authorization (EUA) in the UK, and, subsequently, BNT162b2 received conditional marketing authorizations in Europe (21 December 2020) for active immunization to prevent COVID-19 caused by SARS-CoV-2 [37]. Soon after, many types of vaccines have been approved in multiple countries, such as mRNA, adenovirus-based, protein subunit, and inactivated virus vaccines [38]. Those vaccines had an important impact on reducing the transmission and severity of different SARS-CoV-2 variants in multiple countries, particularly in groups with high-risk populations. However, the global differences in the interrelated variables of population seropositivity and vaccine coverage have widened because of the differences in vaccine strategies (including priority age groups and homologous or heterologous vaccination), vaccine types, and infected populations in different countries [39]. It is hard to quantify the impact of those factors on individual levels due to data unavailability.

We would highlight several limitations in this study. First, some factors potentially correlated with estimates for the severity indicator (e.g., healthcare conditions, climatic factors, economic conditions) were not included in this study due to data unavailability. For example, the shortage of medical resources in some low-income countries brings additional deaths, which lead to an increase in the mortality rate [40]. For example, the shortage of medical resources in some low-income countries brings additional deaths, which lead to an increase in the mortality rate [10]. Second, we only studied cCHR, cCFR, HFR, and HIR to evaluate the severity of SARS-CoV-2 variants. There are other studies of clinical severity using other metrics which were not included in our study. Third, the study data we collected are all from upper- or middle-income countries, including seven studies from Europe, three from North America, and three from Asia. Consequently, it is possible that our findings underestimate the severity of issues in low-income countries. Caution should be exercised when attempting to generalize the results to all ethnicities and nationalities worldwide. Fourth, it is worth noting that the majority of eligible studies included in our review did not take into account preexisting immunity from past infections. It has been observed that prior infection can offer prolonged protection against hospitalization or severe illness [41,42]. This suggests that the disease caused by the Omicron variant may be considerably milder than that caused by Delta at the population level, due to the potential presence of preexisting immunity.

In conclusion, we provide comprehensive estimates of the clinical severity of COVID-19 caused by different SARS-CoV-2 variants based on 13 studies conducted in nine countries. Across all SARS-CoV-2 variants, the Delta and Omicron variants exhibit the highest and lowest severity, respectively, according to the four clinical severity metrics. The results indicate the need for interventions for different SARS-CoV-2 variants and could help with prioritizing variant-specific vaccine development and the formulation of appropriate treatments.

## Figures and Tables

**Table 1:** Characteristics of studies on the clinical severity of COVID-19 caused by SARS-CoV-2 variants included in the meta-analysis.

Study	Location	Variant	Study Period	Population	Vaccine Proportion for Population P_f_ * (%)	Vaccine Proportion for Elderly P_O_ * (%)	cCHR (%)	cCFR(%)	HFR (%)	HIR (%)
Fournier et al. [15]	France	Alpha ^−^	2021.1.18–2021.8.13 18 January 2021–13 August 2021	7894	6.26	78.00	7.56 (6.99, 8.17)	1.66 (1.39, 1.97)	21.94 (18.69, 25.48)	22.78 (19.47, 26.35)
Haas et al. [16]	Israel	Alpha ^#^	24 January 2021–3 April 2021	116,142	5.40	91.47	5.43 (5.31, 5.57)	0.73 (0.69, 0.79)	13.51 (12.70, 14.38)	NR
Twohig et al. [17]	UK	Alpha ^+^	29 March 2021–23 May 2021	34,656	1.31	87.04	2.20 (2.05, 2.36)	NR	NR	NR
Seppälä et al. [18]	Norway	Alpha ^+^	15 April 2021–15 August 2021	13,001	1.59	92.46	2.94 (2.65, 3.24)	0.19 (0.12, 0.28)	6.54 (4.28, 9.51)	NR
Veneti et al. [12]	Norway	Alpha ^+^	3 May 2021–15 August 2021	12,078	1.47	94.26	1.99 (1.75, 2.25)	0.12 (0.07, 0.20)	6.25 (3.54, 10.10)	16.67 (12.18, 22.00)
Butt et al. [11]	Qatar	Beta ^+^	22 March 2021–7 July 2021	451	11.97	NR	19.96 (16.36, 23.95)	0.22 (0.01, 1.23)	1.11 (0.03, 6.04)	15.56 (8.77, 24.72)
Fournier et al. [15]	France	Delta ^−^	18 January 2021–13 August 2021	3730	15.12	78.00	3.54 (2.97, 4.18)	0.70 (0.46, 1.02)	19.70 (13.29, 27.51)	21.21 (14.58, 29.18)
Fisman et al. [19]	Canada	Delta ^+^	7 February 2021–27 June 2021	5989	1.19	60.48	5.83 (5.25, 6.45)	0.67 (0.48, 0.91)	11.46 (8.32, 15.28)	25.79 (21.28, 30.71)
Gupta et al. [20]	India	Delta ^−^	1 March 2021–1 June 2021	677	87.45	12.40	9.90 (7.75, 12.40)	0.44 (0.09, 1.29)	4.48 (0.93, 12.53)	NR
Butt et al. [11]	Qatar	Delta ^+^	22 March 2021–7 July 2021	451	21.06	NR	27.27 (23.21, 31.63)	0.67 (0.14, 1.93)	2.44 (0.51, 6.96)	17.89 (11.56, 25.82)
Twohig et al. [17]	UK	Delta ^+^	29 March 2021–23 May 2021	8682	3.90	87.04	2.26 (1.96, 2.59)	NR	NR	NR
Sheikh et al. [21]	UK	Delta ^+^	1 April 2021–16 August 2021	72,143	11.52	95.56	NR	0.15 (0.12, 0.18)	NR	NR
Sheikh et al. [21]	UK	Delta ^+^	1 April 2021–16 August 2021	71,744	11.52	95.56	NR	0.24 (0.20, 0.28)	NR	NR
Seppälä et al. [18]	Norway	Delta ^+^	15 April 2021–15 August 2021	5430	10.30	94.26	1.55 (1.24, 1.91)	0.09 (0.03, 0.21)	5.95 (1.96, 13.35)	NR
Veneti et al. [12]	Norway	Delta ^+^	3 May 2021–15 August 2021	7977	7.89	94.26	1.34 (1.10, 1.62)	0.06 (0.02, 0.15)	4.67 (1.53, 10.57)	14.95 (8.80, 23.14)
Naleway et al. [22]	USA	Delta ^^^	4 July 2021–25 September 2021	7155	42.05	83.85	6.26 (5.71, 6.85)	1.10 (0.88, 1.37)	17.63 (14.22, 21.49)	24.33 (20.43, 28.58)
Hagan et al. [23]	USA	Delta ^+^	12 July 2021–14 August 2021	172	75.00	81.80	2.33 (0.64, 5.85)	0.58 (0.01, 3.20)	25.00 (0.63, 80.59)	NR
Espenhain et al. [24]	Denmark	Delta ^+^	22 November 2021–7 December 2021	19,137	48.43	98.96	1.52 (1.35, 1.70)	0.07 (0.04, 0.12)	4.80 (2.66, 7.97)	7.59 (4.82, 11.26)
Houhamdi et al. [4]	France	Delta ^−^	28 November 2021–31 December 2021	3075	20.49	88.34	11.93 (10.81, 13.13)	1.27 (0.90, 1.73)	10.67 (7.67, 14.24)	25.61 (21.22, 30.40)
Espenhain et al. [24]	Denmark	Omicron ^+^	22 November 2021–7 December 2021	785	76.31	98.96	1.15 (0.53, 2.17)	0.00 (0.00, 0.47)	0.00 (0.00, 33.62)	11.11 (0.28, 48.25)
Houhamdi et al. [4]	France	Omicron ^−^	28 November 2021–31 December 2021	1119	22.97	88.34	1.88 (1.17, 2.85)	0.09 (0.00, 0.50)	4.76 (0.12, 23.82)	4.76 (0.12, 23.82)

* P_f_ was calculated by dividing the total population of the country by the population who received two doses of COVID-19 vaccine. P_O_ was calculated by dividing the total population of the country by the elderly (>60 years) who received two doses of COVID-19 vaccine. The vaccine data were collected from the cited papers and Our World in Data [25]. NR = not reported. ^+^ indicates that cases were defined as infection with a specific variant of SARS-CoV-2 confirmed via PCR or via whole genome sequencing (WGS); ^−^ indicates that cases were identified via next-generation sequencing (NGS) method; ^#^ indicates that cases were estimated on the basis of swabs tested at Leumit with the TaqPath COVID-19 test (Thermo Fisher Scientific, Pleasanton, CA, USA), which identifies spike gene target failure (SGTF) associated with gene mutations that cause deletions of amino acids 69 and 70 in the spike protein. ^^^ indicates that cases were confirmed in the laboratory, but the identification method was not provided. It should be noted that not all cases were identified using these methods. For the cases without identification, they were identified by the primary variant based on the time period and location.

**Table 2:** Summary of included studies on clinical severity of COVID-19 (cCHR, cCFR, HFR, and HIR) by age group.

Study	Location	Variant	Study Period	Population (Confirmed Cases)	Age Group	cCHR (%)	cCFR (%)	HFR (%)	HIR (%)
Grint et al. [26]	England	Alpha ^+^	16 November 2020–11 January 2021	93,153	all	2.90 (2.80, 3.01)	0.54 (0.49, 0.59)	13.66 (12.39, 15.01)	12.74 (11.50, 14.05)
				22,795	0–24	0.31 (0.24, 0.39)	NR	NR	NR
				35,820	25–44	1.50 (1.38, 1.63)	NR	NR	NR
				27,334	45–64	4.37 (4.13, 4.62)	NR	NR	NR
				7204	≥65	12.47 (11.71, 13.25)	NR	NR	NR
Nyberg et al. [27]	England	Alpha ^+^	23 November 2020–31 January 2021	592,409	all	4.68 (4.62, 4.73)	0.44 (0.42, 0.46)	3.29 (3.08, 3.50)	NR
				31,935	0–24	1.20 (1.15, 1.26)	NR	NR	NR
				63,084	25–44	3.35 (3.28, 3.43)	NR	NR	NR
				115,296	45–64	6.99 (6.87, 7.11)	NR	NR	NR
				118,229	≥65	14.49 (14.17, 14.82)	NR	NR	NR
Veneti et al. [28]	Norway	Alpha ^+^	28 December 2020–2 May 2021	23,169	all	3.82 (3.57, 4.07)	NR	NR	19.91 (17.32, 22.70)
				9915	0–24	0.36 (0.25, 0.50)	NR	NR	NR
				7624	25–44	2.85 (2.48, 3.24)	NR	NR	NR
				1860	45–64	23.17 (21.27, 25.16)	NR	NR	NR
				770	≥65	25.98 (22.91, 29.22)	NR	NR	NR
Haas et al. [16]	Israel	Alpha ^#^	24 January 2021–3 April 2021	116,142	all	5.30 (5.17, 5.43)	0.73 (0.69, 0.79)	13.87 (13.01, 14.75)	NR
				26,818	0–24	2.40 (2.22, 2.59)	0.04 (0.02, 0.07)	1.71 (0.86, 3.04)	NR
				59,594	25–44	2.40 (2.28, 2.53)	0.04 (0.03, 0.06)	1.75 (1.13, 2.57)	NR
				21,843	45–64	8.24 (7.87, 8.61)	6.36 (5.35, 7.51)	7.73 (6.53, 9.06)	NR
				7887	≥65	28.87 (27.87, 29.88)	8.60 (7.99, 9.24)	29.78 (27.90, 31.70)	NR
Veneti et al. [12]	Norway	Alpha ^+^	3 May 2021–15 August 2021	12,078	all	1.99 (1.75, 2.25)	0.12 (0.07, 0.20)	6.25 (3.54, 10.10)	16.67 (12.18, 21.20)
				6292	0–24	0.17 (0.09, 0.31)	NR	NR	NR
				3523	25–44	1.96 (1.53, 2.47)	NR	NR	NR
				2003	45–64	6.00 (5.00, 7.12)	NR	NR	NR
				260	≥65	15.38 (11.22, 20.35)	NR	NR	NR
Veneti et al. [12]	Norway	Beta ^+^	28 December 2020–2 May 2021	548	all	4.20 (2.68, 6.23)	NR	NR	21.74 (7.46, 43.70)
				231	0–24	0 (0, 1.58)	NR	NR	NR
				185	25–44	2.70 (0.88, 6.19)	NR	NR	NR
				118	45–64	12.71 (7.29, 20.10)	NR	NR	NR
				14	≥65	21.43 (4.66, 50.80)	NR	NR	NR
Sheikh et al. [21]	Scotland	Delta ^+^	1 April 2021–16 August 2021	72,143	all	NR	0.15 (0.12, 0.18)	NR	NR
				18,833	0–24	NR	0.03 (0.01, 0.06)	NR	NR
				42,965	25–44	NR	0.05 (0.03, 0.08)	NR	NR
				8137	45–64	NR	0.41 (0.28, 0.57)	NR	NR
				2208	≥65	NR	2.08 (1.53, 2.77)	NR	NR
Sheikh et al. [21]	Scotland	Delta ^+^	1 April 2021–16 August 2021	71,700	all	NR	0.24 (0.20, 0.27)	NR	NR
				13,215	0–24	NR	0.03 (0.01, 0.08)	NR	NR
				34,275	25–44	NR	0.07 (0.05, 0.10)	NR	NR
				18,672	45–64	NR	0.28 (0.21, 0.37)	NR	NR
				5582	≥65	NR	1.56 (1.25, 1.92)	NR	NR
Veneti et al. [12]	Norway	Delta ^+^	3 May 2021–15 August 2021	7977	all	1.34 (1.10, 1.62)	0.06 (0.02, 0.15)	4.67 (1.53, 10.57)	14.95 (8.80, 23.14)
				3964	0–24	0.33 (0.17, 0.56)	NR	NR	NR
				3036	25–44	1.45 (1.05–1.94)	NR	NR	NR
				1057	45–64	3.31 (2.32, 4.58)	NR	NR	NR
				190	≥65	7.89 (4.49, 12.69)	NR	NR	NR

NR = not reported. ^+^ indicates that cases were defined as infection with a specific variant of SARS-CoV-2 confirmed via PCR or via whole genome sequencing (WGS); ^#^ indicates that cases were estimated on the basis of swabs tested at Leumit with the TaqPath COVID-19 test (Thermo Fisher Scientific, Pleasanton, CA, USA), which identifies spike gene target failure (SGTF) associated with gene mutations that cause deletions of amino acids 69 and 70 in the spike protein.

**Table 3:** Statistical results from meta-regression of the effect of the population proportion with two doses of vaccines (P_f_ and P_o_) on clinical severity (cCHR, cCFR, HFR, HIR) for Delta SARS-CoV-2 variant. Each kind of clinical severity was taken as the dependent variable, and each kind of proportion with two doses of vaccines was taken as the predictor. α is the estimated slope of the linear regression with intercept.

Dependent Variable (for Delta Variant)	Predictor	R^2^ (%)	α	*p*-Value	Intercept	*p*-Value
cCHR	P_f_	0.00	0.0319	0.4729	3.6411	0.0729
cCHR	P_O_	15.55	−0.0744	0.1428	10.487	0.0242
cCFR	P_f_	0.00	0.0015	0.8055	0.4199	0.0705
cCFR	P_O_	1.36	−0.0041	0.5118	0.0080	0.1586
HFR	P_f_	0.00	−0.0390	0.6384	11.109	0.0098
HFR	P_O_	0.00	0.0181	0.8312	8.5612	0.2436
HIR	P_f_	5.49	−0.1847	0.3411	24.202	0.0084
HIR	P_O_	25.87	−0.3319	0.1753	48.086	0.0503

## Data Availability

Not applicable.
